# Genetic Analysis of Two Chicken Infectious Anemia Virus Variants-Related* Gyrovirus* in Stray Mice and Dogs: The First Report in China, 2015

**DOI:** 10.1155/2017/6707868

**Published:** 2017-02-23

**Authors:** Lichun Fang, Yang Li, Yixin Wang, Jiayuan Fu, Shuai Cui, Xiaohan Li, Shuang Chang, Peng Zhao

**Affiliations:** College of Animal Science and Veterinary Medicine, Shandong Agricultural University, Tai'an, China

## Abstract

Chicken infectious anemia virus (CIAV) causes acute viral infection in chickens worldwide. It can infect chickens of all ages, but the disease is seen only in young chickens and is characterized by hemorrhagic lesions in the muscles, atrophic changes in the lymphoid organs, aplastic bone marrow, and immunosuppression causing increased mortality. Previous studies have demonstrated that CIAV can be isolated from blood specimens of humans and fecal samples of stray cats. In the present study, two variants of CIAV were isolated from fecal samples of mice (CIAV-Mouse) and stray dogs (CIAV-Dog), respectively. The genome of the two CIAV variants was sequenced and the results of the recombination detection program suggested that the CIAV-Dog strain could be a recombinant viral strain generated from parental CIAV strains, AB119448 and GD-1-12, with high confidence. Particularly, these findings were obtained from the comparison of genetic diversity and the relationship of CIAV between different hosts. This is the first report indicating that there is a significant difference in the number of transcription factor binding sites in CIAV noncoding regions from different hosts. Further studies are required to investigate the large geographic distribution of CIAV and monitor the variants, host range, and associated diseases.

## 1. Introduction

Chicken infectious anemia virus (CIAV) is an economically important pathogen affecting the poultry industry worldwide [1–7]; it was first isolated in Japan in 1979 by Yuasa [8]. The disease is characterized by atrophy of the bone marrow hematopoietic and lymphoid tissues (e.g., thymus) in young chickens, causing severe anemia and immunosuppression leading to increased mortality due to secondary complications [9]. The disease readily spreads horizontally, but vertical transmission appears to be the most important means of dissemination [10, 11]. Horizontal transmission is caused by direct or indirect contact and is most likely to be transmitted orally and through the respiratory tract as well as feces from infected animals [7]. There is no strong evidence of CIAV posing a threat to the human health. However, existing evidence suggests that CIAV-related viruses can be found in feces of stray cats, and feces, blood, and skin of humans [12–18], indicating that CIAV may be a potential threat to human health.

CIAV is a member of the genus* Gyrovirus* belonging to the family Circoviridae [3]. The virion of CIAV is nonenveloped, icosahedral, and approximately 25 nm in diameter, with a negative-sense, single-stranded, circular DNA genome, approximately 2298–2319 nucleotides in length [4]. The genome consists of three major partially overlapping open reading frames that encode peptides of 51.6 (VP1), 24 (VP2), and 13.6 (VP3) kDa [19]. The noncoding region of the CIAV genome is only 0.3 kb, but it shows complete promoter activity and contains more than a dozen conserved sequences related to replication and transcriptional regulation [20].

CIAV isolates show extremely limited genetic variability worldwide [21]. Viruses belonging to the Circoviridae family are not well studied, and little is known about CIAV recombination events. Cases of CIAV recombination have been reported in chickens in China, and the only recombination region is located in the coding region of VP1 [22, 23]. Zhang et al. reported the first evidence of CIAV homologous recombination in cats [17].

In this study, two strains of CIAV from stray dogs and mice were isolated for the first time in an attempt to elucidate the mechanism of CIAV infection in various species. We analyzed the genetic diversity among different hosts of CIAV. The first evidence of CIAV homologous recombination in dogs is explored and an obvious difference in the number of transcription factor binding sites between different hosts was found.

## 2. Materials and Methods

### 2.1. Material Source

During the year 2015, fecal samples from 42 stray dogs and 50 mice were collected in Tai'an, Shandong.

### 2.2. DNA Extraction and CIAV Detection

DNA was extracted from fecal samples of 92 subjects using a commercial TIANamp Genomic DNA Kit (Tiangen Biotech Co., Ltd., China) according to the manufacturer's instructions. DNA extracted from all the samples was stored at −20°C until further analysis. Primers were designed using Primer Premier 6.0 to amplify 842 bp of the partial coding regions of CIAV. The forward primer was 5′-GCATTCCGAGTGGTTACTATTCC-3′ and the reverse primer was 5′-CGTCTTGCCATCTTACAGTCTTAT-3′. The PCR reaction was performed in a total volume of 25 *μ*L containing 2.5 *μ*L PCR buffer (Mg^2+^), 2 *μ*L dNTPs (2.5 mM), 0.5 *μ*L of each primer, 17 *μ*L distilled water, 1 *μ*L template DNA, and 0.5 *μ*L rTaq DNA polymerase (TaKaRa, Biotechnology, Co. Ltd., Dalian, China). Amplification for CIAV was performed as follows: predenaturation at 95°C for 5 min, followed by 30 cycles of denaturation at 95°C for 30 sec, annealing at 55°C for 50 sec, and extension at 72°C for 1 min. PCR products of 842 bp were analyzed using 1% agarose gel electrophoresis. Positive and negative controls were included in each PCR.

### 2.3. Virus Isolation

The two CIAV positive samples were propagated in Marek's disease virus-transformed MDCC-MSB1 cell line. First, 0.5 mL virus suspension was mixed with the MSB1 cell pellet resuspended in 0.5 mL RPMI 1640 medium and incubated at 37°C for 1 h. Then, 5 mL RPMI 1640 medium was added to the mixture, and the cells were suspended and incubated at 37°C for 3 d. One-milliliter cell suspension was transferred to 4 mL fresh RPMI 1640 medium followed by incubation. Cells were repeatedly passaged and the virus was isolated.

### 2.4. Amplification, Cloning, and Sequencing of the CIAV Genome

Based on the CIAV sequences published in GenBank (Table 1), three pairs of primers were designed using DNAStar 7.0, and total DNA obtained in the previous step was amplified using PCR (Table 2). The amplified fragments were 843 bp, 989 bp, and 802 bp in length, to cover the entire CIAV genome. The PCR amplification was carried out in 50 *μ*L total volume containing 25-*μ*L buffer I, 16 *μ*L dNTPs, 0.5 *μ*L of each primer, 13.5 *μ*L distilled water, 1 *μ*L DNA, and 0.5 *μ*L LA Taq polymerase (TaKaRa, Biotechnology, Dalian, China). All PCR amplification products were analyzed via agarose gel electrophoresis, followed by ethidium bromide staining. The PCR products were purified with a Gel Band Purification Kit (Omega Bio-Tek, USA) and cloned into the pMD19-T vector (TaKaRa Bio Inc., Japan) and sequenced using an ABI 3730 Sanger-based genetic analyzer (Carlsbad, CA, USA). Sequencing was performed in triplicate.

### 2.5. Whole Genome Sequence Alignment and Phylogenetic Analysis

To establish the genotypes and clusters of the sequenced CIAV variants in this study, phylogenetic analysis was performed based on the complete genome sequence. The complete nucleotide sequence of CIAV variants and reference sequences were obtained from GenBank (Table 1). The DNA sequences and amino acid sequences (VP1, VP2, and VP3) were assembled using DNAStar (version 7; Madison, WI, USA). Multiple-sequence alignment was performed using Clustal W (BioEdit version 7). A neighbor-joining (NJ) tree based on the full-length nucleic acid sequence was constructed using MEGA 5.1 program [24]. The robustness of the NJ tree was evaluated via a bootstrap analysis with 1000 replicates.

### 2.6. Sequence Analysis of the CIAV Genome in Noncoding Region

Based on the entire aligned genome, the homology of the noncoding region was analyzed using DNAStar software to compare the homology of CIAV with that of different reference strains. According to a previous report, CIAV binding factors and consensus sequences of the binding sites [20] in different hosts were searched and noted (Table 3).

### 2.7. Identification of CIAV Genomic Recombination

To detect recombinant patterns, parental strains, and potential putative recombination breakpoints in CIAV variants in this study, the recombination detection program 4 (RDP 4) was applied [25] using nine methods (RDP, GENECONV, BootScan, MaxChi, Chimaera, SiScan, Phyl- Pro, LARD, and 3Seq) with general settings (window size = 20, highest multiple-comparison-corrected *P* value = 0.01, Bonferroni correction, finding consensus daughter sequences, and polishing breakpoints). For the RDP algorithm, the reference sequence parameter (internal and external reference) was used as recommended in the manual.

To confirm the potential parental CIAV lineages and putative recombination breakpoints previously analyzed and estimated by RDP software, SIMPLOT software v. 3.5.1 [26] was employed to further investigate this possible recombination event.

## 3. Results

### 3.1. Detection of Viruses and Amplification of the Whole Genome by PCR

PCR analysis was performed on fecal samples from 92 subjects (42 stray dogs and 50 mice); two variants of CIAV were confirmed and named as CIAV-Dog and CIAV-Mouse, respectively. The whole genome of CIAV was amplified using three sets of primers, and the genome was further analyzed using DNA sequencing.

### 3.2. Complete Sequence and Phylogenetic Analysis

The complete genome sequences of the two CIAV variants were submitted to GenBank, under the accession numbers KU645525 and KU645524. The length of the genome of both CIAV-Mouse and CIAV-Dog strains was 2298 bp. The whole genome sequence of these two strains was compared to that of other 35 strains from different countries and different hosts in GenBank (Table 1). According to the database, CIAV-Dog strain showed the highest homology (98.2%) with SD22 strain (accession number DQ141673) isolated in Shandong, China, while it showed the lowest homology (40.4%) with Hong Kong Human GyV4 strain (accession number JX310702). CIAV-Mouse strain showed the highest homology (98.3%) with Australia 704 (accession number CAU65414) and Japan TR20 strains (accession number AB027470), while similar to CIAV-Dog, CIAV-Mouse showed the lowest homology (40.9%) with Hong Kong Human GyV4 strain (accession number JX310702).

**Figure 1:**
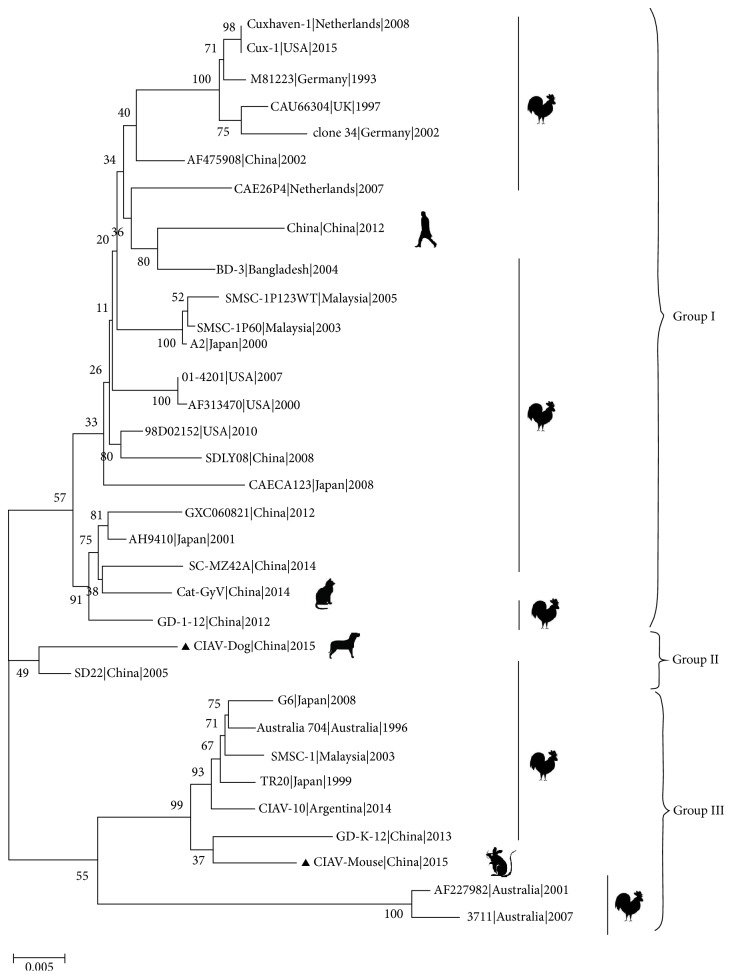
The molecular genetic evolution tree of CIAV strains based on the whole genome nucleotide sequence. Sequences from the present study are named as CIAV-Dog and CIAV-Mouse which are shown with a “black triangle.” GenBank sequences were given the strains name followed by country name and time. The three major groups were identified as Group I, Group II, and Group III. The whole sequences were analyzed by using MEGA5.1 software with neighbor-joining (NJ) phylogenetic tree methods together with the novel sequence. Each tree was produced using a consensus of 1000 bootstrap replicates.

A phylogenetic analysis was performed using the two novel CIAV sequences and the 31 full-length genome sequences obtained from the public database based on the whole genome nucleotide sequence (Figure 1). As seen in Figure 1, the evolutionary tree can be divided into three groups of genes. These genes contain most of the CIAV strains in the first group, including the Cat-Gyv strain, which came from different hosts of different countries in different years. This group can be divided into 2 clusters. The China strain from humans was included in the first small cluster of branches, while the Cat-Gyv strain was included in the second small cluster of branches.

CIAV-Dog and SD22 strains isolated in 2005 were in the second group. The third group contained nine genomic sequences, including CIAV-Mouse. GD-K-12 strain was isolated in China in 2013. CIAV-10 strain was isolated from Argentina in 2014. TR20 and G6 strains were isolated in Japan in 1999 and 2008, respectively. The three strains (Australia 704, AF227982, and 3711) were isolated in Australia in 1996, 2001, and 2007, respectively. SMSC-1 was isolated in Malaysia in 2003. As stated above, the strains can be distinctly divided into 3 clusters in this group. The isolates from Australia (AF227982 and 3711) isolated in 2001 and 2007, respectively, were included in the first small cluster of branches; CIAV-Mouse and GD-K-12 isolated in 2013 were included in the second small cluster of branches, and the other five strains were included in the third small cluster of branches. The Chinese isolate, GD-K-12, isolated in 2013 had a lower growing ability and transmission capacity [2]. We speculated that CIAV-Mouse might also be an attenuated strain.

### 3.3. Sequence Analysis of CIAV Genome in Noncoding Region

**Figure 2:**
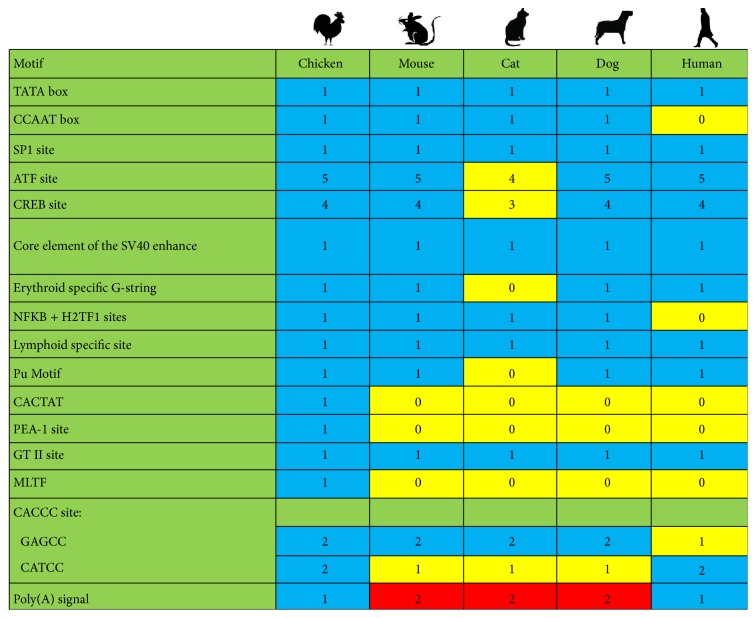
The number of transcription factor binding sites in noncoding regions of different hosts. Based on the whole genome that has been aligned. The transcription factor binding sites were searched and counted. The blue label means the motif numbers are same with chicken. The yellow label means deletion. The red label means addition.

As described in Table 3, we compared the noncoding region of the genome regulation related motif with that of other reference strains in different hosts (Figure 2).

The noncoding sequences in the CIAV genome were highly conservative (nucleotide homology 90.0–99.8%), especially the transcription factor binding sites. These transcription factor binding sites from the CIAV in chickens were similar, but there existed an obvious difference in individual motifs among viruses from different hosts; compared to the CIAV in chicken, CIAV from other hosts lack the transcriptional binding site-related motif. The CIAV variants isolated from mice, cats, dogs, and humans do not contain CACTAT, PEA-1, and MLTF motifs, but the CIAV strains isolated from chicken contain all the three motifs (CACTAT, PEA-1, and MLTF). Interestingly, there are two poly(A) signals in all CIAV stemming from cats, dogs, and mice; whether there exist different terminations in the transcription process or they enhanced the stability of mRNA remains to be studied. All these differences can be seen in Figure 2.

### 3.4. Recombination Analysis

Recombination analysis identified 34 CIAV sequences from different hosts and detected one significant recombination event using the RDP 4 software. For this event, seven out of nine algorithms detected significant recombination at the same location in the CIAV genome with *P* values ranging from [8.629 × 10^−09^–1.174 × 10^−04^] (Table 4). The location of two significant break points was in the VP1 coding region (nt positions 1686 and 2122) of CIAV-Dog, which was considered as the daughter or recombinant, with the major parent being the GD-1-12 and the minor parent being AB119448.

**Figure 3:**
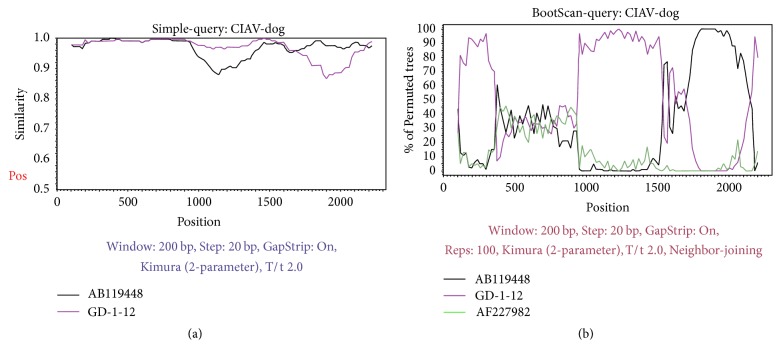
SimPlot recombination analysis. (a) Similarity plot generated using the complete genome of CIAV-Dog as query sequence, GD-1-12 (purple) and AB119448 (black) as two parent groups. Kimura 2-parameter was used as distance model with a transition-transversion ratio of 2. The *Y*-axis refers to percentage identity with a sliding window size of 200 bp and with a step size of 20 bp between plots. The *X*-axis represents the nucleotide positions in alignment. (b) Bootscanning analysis performed with CIAV-Dog as query sequence using a sliding window of 200 nt moving in 20 nt steps.

In addition, to find proof for the results obtained using RDP software, a similarity plot analysis was carried out using SimPlot software. The analysis of the recombination was corroborative with the results of RDP software analysis. The results indicated that the recombinant exhibited a high nucleotide similarity with the isolates GD-1-12 (purple) and AB119448 (black) (Figure 3(a)). Bootscanning analysis also executed and confirmed the recombination event (Figure 3(b)).

## 4. Discussion

CIAV was first reported by Yuasa et al., and since then the infection is found to be very common in chickens worldwide. In this study, two strains of CIAV from dogs and mice were first isolated, reported, and named as CIAV-Dog and CIAV-Mouse, respectively, and their whole genomes were sequenced. A previous study showed that CIAV has been identified in two different hosts, including cats and humans [17]. A high prevalence of CIAV DNA was confirmed in diarrheal and normal feces from Chilean children [14]. Phan et al. reported that the high rate of codetection of three* Gyroviruses* (CIAV, AGV2/HGV, and GyV3) in human specimens might indicate common dietary exposure to foods that contain all the three viruses (e.g., chicken skin and meat) [14]. This may imply some uncertain dependency or interdependence on the proliferation of success. Although the results of this study found that CIAV variants were isolated from mouse and dog, they do not testify to the change of their natural host. All these variants share a common characteristic—a very high sequence similarity with CIAV from chickens. This finding indicates that the CIAV variant might have originated from CIAV-infected chickens. We know that these potential hosts are not the natural hosts of CIAV. However, it has been shown experimentally that the two hosts (dogs and mice) are carriers of CIAV. Davidson et al. also reported that feathers contribute to the horizontal transmission of CIAV, by carrying CIAV either on their surface or within the feather pulp [27]. The study has proved that dogs, mice, cats, or humans are unlikely to be involved in the epidemiology of CIAV. These potential hosts have the characteristics of mobility; thus they potentially boost CIAV transmission if they are carrying the virus. The results of this study revealed the expanded host range and rich genetic diversity of CIAV and highlighted the potential threat of CIAV to the health of mammals.

Moreover, we also tried to explore molecular characteristics of CIAV by analyzing the noncoding regions. The noncoding region of CIAV genome is only 0.3 kb, but it showed a complete promoter activity [20]. In the corresponding expression vectors and specific cells, the complete noncoding region of CIAV genome can stimulate the expression of human growth hormone (hGH) gene [28]. In the noncoding region of CIAV, approximately 300 nucleotides are centrally distributed with more than a dozen conserved sequences related to replication and transcriptional regulation [20]. Whether these conserved sequences are necessary for the replication of the virus or for maintaining a balance between the virus and the specific cell is unclear and further studies are still required. In different hosts, the noncoding region sequences are highly conserved, but analysis of transcription factor binding sites shows obvious difference between the hosts (Figure 2). Whether the deletion or addition of the regulatory sites is due to the change in host or the mutation sites made it easier to spread to different hosts needs to be studied further. However, we assume that this obvious difference could be a way to estimate the variation among viruses in different hosts.

Natural recombination in VP1 of the CIAV genome, with a resultant new genotype, suggests a faster CIAV evolution [22]. A high mutation and gene recombination rate play an important role in the evolution of viruses, which is also the main cause of genetic diversity. In the process of restructuring, a large number of sequence changes can occur through genetic information exchange with other related viruses. Additionally, through the recombination analysis, there was a very high rate to support that CIAV-Dog might be a recombinant of AB119448 and GD-1-12 stemmed from chicken. Moreover, the recombination did not change the open reading frame. These results indicate that the CIAV variant might have originated from CIAV-infected chickens. Homologous recombination might cause a potential change in viral epidemiology. It is important to understand the extent of sequence variability in CIAV, to improve the management strategies to prevent CIAV infections in chickens, and to prevent the transmission from chickens to humans or other hosts. In view of the high infection rate of CIAV, the analysis of the pathogenicity of the recombinant virus in different hosts will be of great significance in future research.

In conclusion, we reported that chickens are the natural hosts of CIAV, but CIAV variants were detected in cats, dogs, mice, and humans. To explore the potential reason for increased transmission of CIAV, we should focus on the possibility of CIAV transmission by chickens, mice, cats, dogs, and humans. Considering the genetic evolution of CIAV strains, they have no apparent relationship with time and geographical area, and homologous recombination of CIAV can occur in different hosts. The present study noted that extensive surveillance of the virus in poultry, mammals, and other hosts should be carried out in the future.

## Figures and Tables

**Table 1:** The GenBank accession numbers of full-length CIAV genomes in isolates from different hosts.

Accession number	Strain name	Host	Year	Country (area)	Whole length
M81223	M81223	Chicken	1993	Germany	2298 bp
CAU65414	Australia 704	Chicken	1996	Australia	2298 bp
CAU66304	CAU66304	*Gallusgallus*	1997	UK	2319 bp
AB027470	TR20	Chicken	1999	Japan	2298 bp
AB031296	A2	Chicken	2000	Japan	2298 bp
AF313470	AF313470	Chicken	2000	USA	2294 bp
AF227982	AF227982	Chicken	2001	Australia	2286 bp
AB046590	AH9410	Chicken	2001	Japan	2298 bp
AF475908	AF475908	Chicken	2002	China	2298 bp
AJ297685	Clone 34	Chicken	2002	Germany	2297 bp
AF390102	SMSC-1P60	Chicken	2003	Malaysia	2298 bp
AF285882	SMSC-1	Chicken	2003	Malaysia	2298 bp
AF395114	BD-3	Chicken	2004	Bangladesh	2298 bp
DQ141673	SD22	Chicken	2005	China	2298 bp
DQ217401	SMSC-1P123WT	Chicken	2005	Malaysia	2298 bp
D10068	CAE26P4	Chicken	2007	Netherlands	2298 bp
EF683159	3711	Chicken	2007	Australia	2279 bp
DQ991394	01-4201	Chicken	2007	USA	2298 bp
M55918	Cuxhaven-1	Chicken	2008	Netherlands	2319 bp
FJ172347	SDLY08	Broiler chicken	2008	China	2298 bp
D31965	CAECA123	Chicken	2008	Japan	2319 bp
AB119448	G6	Chicken	2009	Japan	2298 bp
AF311892	98D02152	Chicken	2010	USA	2298 bp
JX260426	GD-1-12	Chicken	2012	China	2298 bp
JX964755	GXC060821	Chicken	2012	China	2292 bp
JQ690762	China	Human	2012	China	2316 bp
KF224935	GD-K-12	Chicken	2013	China	2298 bp
KJ872513	CIAV-10	Chicken	2014	Argentina	2298 bp
KM496307	SC-MZ42A	Chicken	2014	China	2298 bp
NC001427	Cux-1	Chicken	1991	USA	2319 bp
KC414026	Cat-Gyv	Cat	2014	China	2295 bp
JQ308210	GyV3	Human	2011	USA	2359 bp
JX310702	GyV4	Human	2012	Hong Kong	2034 bp
KU645524	CIAV-Dog	Dog	2015	China	2298 bp
KU645525	CIAV-Mouse	Mouse	2015	China	2298 bp

**Table 2:** Primers used for genome amplification.

Primers	Sequence	Product length
F1	5′-GCATTCCGAGTGGTTACTATTCC-3′	843 bp
R1	5′-CGTCTTGCCATCTTACAGTCTTAT-3′	
F2	5′-CGAGTACAGGGTAAGCGAGCTAAA-3′	989 bp
R2	5′-TGCTATTCATGCAGCGGACTT-3′	
F3	5′-ACGAGCAACAGTACCCTGCTAT-3′	802 bp
R3	5′-CTGTACATGCTCCACTCGTT-3′	

**Table 3:** Transcription factor-binding sequence elements^A^.

Motif	Consensus sequence	CIAV sequence	Numbers of sites
TATA box	GTATA(A/T)A(A/T)	TATATAT	1
CCAAT box	AGCCAAT	AGCCAAT	1
SP1 site	GGGCGG	GGGCGG	1
ATF site	ACGTCA	ACGTCA	5
CREB site	(T/G)(T/A)CGTCA	TACGTCA	4
Core element of the SV40 enhancer	GTGG(A/T)(A/T)(A/T)	GTGGTTA	1
Erythroid specific G-string	GGGGGGGGGG	GGGGGGGGGG	1
NFKB + H2TF1 sites	GGGGATTCCCC	GGGGATTCCCC	1
Lymphoid specific site	CTATTC	CTATTC	1
Pu Motif	9 purines	GAAAAGGGGGGGGGG	1
CACTAT	AT rich CACTAT	CACTAT	1
PEA-1 site	GGAAGTGACTAAC	GAAAGTGACTTTC	1
GT II site	G(G/C)TGTGGAA(A/T)GT	CGTTGCGAAAGT	1
MLTF	GGCCACGTGACC	TGCCACTGTCGA	1
CACCC site:	CACCC	CAGCC	2
		CATCC	2
Poly(A) signal	AATAAA	AATAAA	1

^A^Binding factors and consensus sequences of the binding sites are reviewed in references [29, 30].

**Table 4:** *P* values of the recombinant calculated by different methods embedded in the RDP 4 software package.

Methods	Av. *P* Val
RDP	1.174 × 10^−04^
GENECONV	2.323 × 10^−05^
BootScan	2.262 × 10^−04^
MaxChi	5.023 × 10^−05^
Chimaera	2.014 × 10^−07^
SiScan	1.220 × 10^−06^
PhyIPro	n/a
LARD	n/a
3Seq	8.629 × 10^−09^
